# Quality Assessment of Videos About Dengue Fever on Douyin: Cross-Sectional Study

**DOI:** 10.2196/76474

**Published:** 2025-09-26

**Authors:** Youlian Zhou, Liang Yang, Li Luo, Lianghai Cao, Jun Qiu

**Affiliations:** 1 Intensive Care Unit, Chengdu Integrated TCM & Western Medicine Hospital, 18 North Vientiane Road, Chengdu, 610016, China, 86 18380215259

**Keywords:** dengue fever, douyin, DISCERN, Journal of American Medical Association, Global Quality Score, video quality

## Abstract

**Background:**

Dengue fever has evolved into a significant public health concern. In recent years, short-video platforms such as Douyin have emerged as prominent media for the dissemination of health education content. Nevertheless, there is a paucity of research investigating the quality of health education content on Douyin.

**Objective:**

This study aimed to evaluate the quality of dengue videos on Douyin.

**Methods:**

A comprehensive collection of short videos pertaining to dengue fever was retrieved from the popular social media platform, Douyin, at a designated point in time. A systematic analysis was then performed to extract the characteristics of these videos. To ensure a comprehensive evaluation, three distinct scoring tools were used: the DISCERN scoring tool, the JAMA benchmarking criteria, and the GQS method. Subsequently, an in-depth investigation was undertaken into the relationship between video features and quality.

**Results:**

A total of 156 videos were included in the analysis, 81 of which (51.9%) were posted by physicians, constituting the most active category of contributor. The selected videos pertaining to dengue fever received a total of 718,228 likes and 126,400 comments. The video sources were categorized into four distinct classifications: news agencies, organizations, physicians, and individuals. Individuals obtained the highest number of video likes, comments, and saves. However, the findings of the study demonstrated that physicians, organizations, and news agencies posted videos are of higher quality when compared with individuals. The integrity of the video content was analyzed, and the results showed a higher percentage of videos received a score of zero points for outcomes, management, and assessment, with 69 (45%), 57 (37%), and 41 (26%), respectively. The median Total DISCERN scores, JAMA, and GQS of the 156 dengue-related videos under consideration were 26 (out of a total of 80 points), 2 (out of a total of 4 points), and 3 (out of a total of 5 points), respectively. Spearman correlation analysis was conducted, revealing a positive correlation between video duration and video quality. Conversely, a negative correlation was observed between the following variables: video comments and video quality, and the number of days since posting and video quality.

**Conclusions:**

This study demonstrates that the quality of short dengue-related health information videos on Douyin is substandard. Videos uploaded by medical professionals were among the highest in terms of quality, yet their videos were not as popular. It is recommended that in future, physicians employ more accessible language incorporating visual elements to enhance the appeal and dissemination of their videos. Future research could explore how to achieve a balance between professionalism and entertainment to promote user acceptance of high-quality content. Moreover, platforms may consider employing algorithmic optimization or content recommendation mechanisms to encourage users to access and engage with more high-quality health science videos.

## Introduction

The dengue virus (DENV) is considered to be among the most significant mosquito-borne pathogens, and it was listed among the top 10 global health threats in 2019 [1]. The dengue virus can cause a wide range of clinical manifestations, varying from a relatively minor illness characterized by flu-like symptoms and known as dengue fever (DF) to a condition that carries a significant risk of mortality and is known as dengue shock syndrome (DSS). The 2009 World Health Organization (WHO) classification system for dengue is predicated on the levels of severity. The system includes the following classifications: dengue without warning signs, dengue with warning signs (which includes abdominal pain, persistent vomiting, fluid accumulation, mucosal bleeding, lethargy, liver enlargement, and increasing hematocrit with decreasing platelets), and severe dengue, which includes dengue with severe plasma leakage, severe bleeding, or organ failure [2]. A 30-fold increase in dengue incidence has been seen in the last 50 years, and more than 50% of the world’s population, in over 100 countries, is living in areas at risk of DENV infection [3,4]. It is evident that in 2023, the number of reported infections worldwide exceeded five million, resulting in approximately five thousand deaths, which approximates a historic peak [5]. These data indicate that dengue fever is a significant global public health concern, necessitating the implementation of effective control and prevention strategies.

The emergence of dengue fever in China poses a considerable risk to public health and social stability. An outbreak of dengue fever occurred in Guangdong in 1978, resulting in a total of 22,122 cases of infection and 16 deaths. Since then, dengue has been identified annually, with outbreaks occurring intermittently at 4‐7-year intervals [6,7]. A comprehensive review of the extant literature reveals that from 1978 to 2019, dengue fever outbreaks led to a total of 747,417 cases and 622 deaths [1]. These alarming figures not only reflect the widespread spread of dengue fever in the country, but also reveal the challenges faced in preventing and controlling the disease. The implementation of efficacious prevention and control strategies is imperative to mitigate the societal burden of dengue fever and to protect public health and safety.

The outpatient management of dengue fever by primary care physicians represents the primary treatment strategy, as the majority of cases present as mild and self-limiting [8]. It is standard practice to advise patients with dengue who are being treated on an outpatient basis to seek immediate medical assistance when warning signs of dengue are present. The prevention of dengue virus infection requires the acquisition of knowledge, the development of favorable attitudes and the adoption of appropriate practices among the general public [9]. The absence of awareness regarding the transmission of the dengue infection and the preventive measures that can be employed to avoid infection may elevate the risk of disease transmission. A study conducted in China demonstrated that the Dai people possess a comprehensive understanding of dengue fever. However, their perceptions of disease susceptibility and severity are relatively low [10]. Recent research has demonstrated that, despite the efforts of physicians to educate their patients about warning signs and their significance, many patients either lack recall or fail to fully comprehend these messages. Consequently, it is imperative to enhance public cognizance and comprehension of dengue fever and to furnish precise counsel on prevention. This is an indispensable strategy for controlling and preventing the dissemination of dengue fever.

The dissemination of information through various forms of mass media, including official WeChat accounts, print media, leaflets, broadcast media, and the Internet, directly influenced the public’s knowledge about dengue fever and their actions regarding mosquito control [11]. As indicated in the 50th Statistical Report on China’s Internet Development Status, published by the China Internet Network Information Center, the nation’s Internet penetration rate was 74.45% as of June 2022, with 1.051 billion Internet users [12,13]. The number of users of short videos has increased markedly in recent years, reaching approximately 962 million users across the country [12]. TikTok, a video-sharing medium, has amassed a user base of 600 million in China alone [14]. It has been reported that as of July 2020, TikTok’s videos pertaining to the novel coronavirus were viewed a total of 93.1 billion times during the ongoing pandemic [15]. Nevertheless, a considerable body of research has demonstrated that approximately half of the videos shared on the social media platform TikTok disseminate misinformation or present a deficient level of expertise [16,17].

A substantial number of videos related to dengue fever were identified on the Chinese version of the video-sharing platform TikTok. Nevertheless, the reliability of the information provided remains questionable. The objective of this study was to evaluate the quality of information presented in videos related to dengue fever that are available on the Chinese version of the social media platform.

## Methods

### Data Collection

In this cross-sectional study, the Chinese version of TikTok, known as Douyin, was queried on November 23, 2024, for the specific term “dengue fever” in Chinese. The videos were sorted by a comprehensive ranking with the publishing time set to unlimited, which represents the default setting on TikTok. This analysis focused on videos that addressed dengue fever directly. However, videos pertaining to other topics, in addition to commercials, videos in languages other than Chinese, duplicate videos, videos of personal experiences and videos lacking audio, were excluded from the study. A total of 324 videos pertaining to dengue fever were identified on the Douyin platform, of which 156 were selected for further data extraction and analysis following a rigorous screening process.

The following characteristics of each Douyin video pertaining to dengue fever were recorded and subsequently subjected to a comprehensive analysis, including the title, content, quantity of likes and comments, number of saves, days since upload, duration of video, and status of video source, such as the number of fans and likes for the uploader. All relevant data were stored in a Microsoft Excel spreadsheet.

### Classification of Videos

Video sources were classified into the following categories: (1) News agencies, including network platforms, print media, television, and radio stations; (2) Organization entities such as healthcare facilities, public health agencies, research teams, and academic institutions; (3) Physicians; and (4) Individuals, especially those without professional medical qualifications.

### Quality Assessment

The quality of the video content was evaluated using three distinct assessment instruments: the DISCERN instrument (Table S1 in Multimedia Appendix 1), the benchmark criteria established by the Journal of the American Medical Association (JAMA) (Table S2 in Multimedia Appendix 1), and the Global Quality Scores (GQS) (Table S3 in Multimedia Appendix 1). A systematic review indicates that, since its publication in 1998, DISCERN has become one of the most frequently employed instruments for evaluating the quality of health information [18]. In recent times, it has been utilized to assess the quality of information conveyed in health education videos [19,20]. The instrument is comprised of 16 items, with response options anchored on a 5-point rating scale, ranging from 1 (indicating poor performance) to 5 (indicating good performance). The 16-item scale is divided into three sections. The first section concerns the reliability of the publication and comprises eight items. The second section evaluates the quality of information regarding treatment choices and comprises seven items. The third section requests an overall rating of the publication, which is based on a single item [21]. The DISCERN scores spanned from 16 to 80, with classifications as follows: very poor (16-26), poor (27-38), fair (39-50), good (51-62), and excellent (63-80) [22].

The JAMA benchmark criteria were employed to evaluate the reliability of the video source. The aforementioned criteria are constituted of four individual criteria, which are as follows: (1) providing authorship; (2) listing copyright information and references, as well as sources of content; (3) providing the initial date and subsequent updates; and (4) disclosing conflicts of interest, funding, sponsorship, advertising support, and video ownership. The total possible score is four points, with each criterion accounting for one point, respectively [23]. Furthermore, the GQS was utilized to assess the quality of the educational content, with a specific focus on evaluating the quality of the material, the comprehensiveness of the information presented, and its utility for patients. The instrument yielded scores on a scale ranging from one (which indicates poor quality) to five (which indicates excellent quality and flow) [21]. Jun Qiu and Youlian Zhou, both Master of Medicine and critical care medicine department members, served as the raters. The determination of the scores was conducted through a process of discussion, and an arbitrator, Liang Yang, a highly qualified critical care physician, was appointed to resolve conflicts between raters and to issue the final scores. Subsequently, all authors achieved a consensus on the ratings.

### Completeness of Content

In order to evaluate the completeness of the video content, six questions from the study conducted by Goobie et al were adopted [24]. The aforementioned six questions inquire as to the extent to which a given video addresses the following elements: definitions of diseases, their signs and symptoms, risk factors, evaluations, management strategies, and outcomes. Each component was evaluated using a three-point scale, with a value of zero assigned to those that were not addressed, one to those that were partially addressed, and two to those that were fully addressed.

### Statistical Analyses

In cases of categorical variables, the reported values are the count and percentage. The distribution of continuous variables does not align with the normality assumption. To address this, we express the descriptive statistics for continuous variables using the median and interquartile range. The Kruskal-Walli’s test was employed to evaluate the existence of statistically significant differences between multiple groups. Subsequently, the Dunn multiple comparisons test was utilized to facilitate intergroup comparisons. The interobserver agreements were assessed using intraclass correlation coefficients (ICC). The relationships between the quantitative variables were evaluated using Spearman correlation analysis. A *P* value of less than .05 was deemed to be statistically significant. The data were analyzed using the SPSS (version 27; IBM Corp) software and GraphPad Prism (version 10 for Mac).

### Ethical Considerations

No human biospecimens or experimental organisms were used in this study. As the study focused only on digital content analysis, the institutional ethics committee did not consider it necessary to conduct a review.

## Results

### Video Sources

A total of 156 videos were included in the analysis, 81 (51.9%) were posted by physicians, constituting the most active category of contributor. News agencies were the second most active category, posting 34 (21.8%) videos. Individuals posted 33 (21.2%) videos, while organizations posted the fewest, with 8 (5.1%) videos (Table 1).

**Table 1.** Distribution of video sources.

Video sources	Values, n (%)
Physicians	81 (51.9)
News agencies	34 (21.8)
Individuals	33 (21.2)
Organizations	8 (5.1)

### Basic Characteristics of Videos

The selected video pertaining to dengue fever received a total of 718,228 likes and 126,400 comments. The most recent video relevant to this subject was uploaded one day before the collection of data, while the oldest was uploaded 1190 days prior to data collection. The average number of days since upload was 89 (SD166). The duration of the videos exhibited significant variation, with the longest video lasting 421 seconds and the shortest video lasting 12 seconds. The mean video duration was 86 (SD 66) seconds. The highest number of fans and likes for the uploader was observed for news agencies, while the lowest was recorded for organizations. Individuals obtained the highest number of video likes, comments, and saves. The number of days since video was uploaded, and the length of the video, were longest for individuals, followed by physicians and news agencies, and lastly, organizations (see Table 2).

**Table 2.** Basic characteristics for Douyin of different sources.

Video sources	Account fans	Account likes	Video likes	Video comment	Video saves	Days since upload (d)	Duration (sec)
News agencies, median (IQR)	386500.00 (69250.00-2827500.00)	3997500.00 (365500.00-48277500.00)	328.00 (129.00-2894.50)	19.50 (1.50-243.50)	99.00 (12.25-619.50)	27.54 (12.39-134.47)	57.00 (28.00-102.75)
Organizations, median (IQR)	17500.00 (1900.75-42000.00)	125500.00 (17145.75- 348000.00)	102.00 (19.75-821.50)	41.00 (1.75-222.25)	22.00 (7.50-200.75)	21.96 (15.10-46.39)	55.00 (19.50-78.00)
Physicians, median (IQR)	42000.00 (13000.00- 598000.00)	334000.00 (71000.00- 3704000.00)	413.00 (52.00-1990.00)	30.00 (3.00-127.00)	77.00 (16.00-400.00)	26.29 (16.17-69.31)	71.00 (47.00-92.00)
Individuals, median (IQR)	22000.00 (3720.00- 156000.00)	189000.00 (22000.00-1964000.00)	668.00 (176.00-1860.00)	97.00 (26.00-361.00)	114.00 (39.00-299.00)	44.07 (18.41-210.27)	85.00 (40.00-110.00)

### Content of Videos

**Figure 1.**
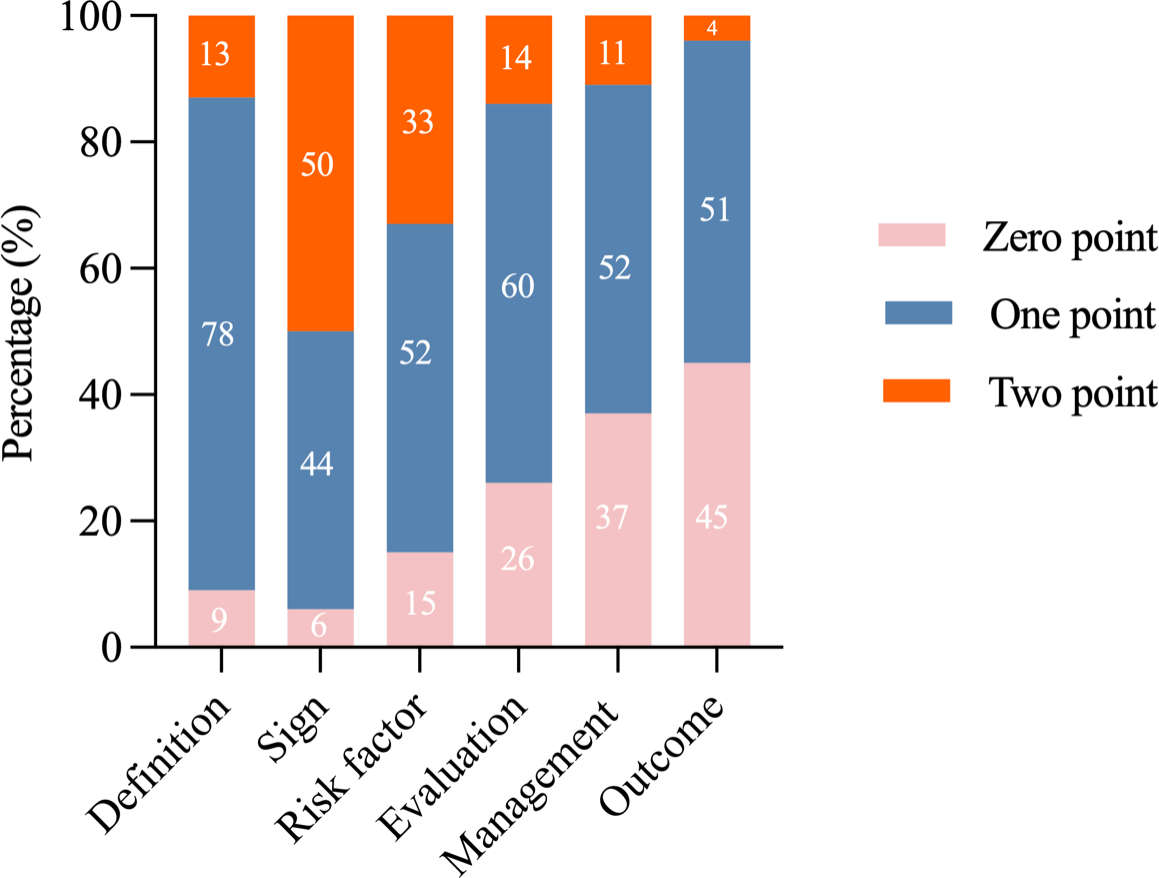
Percentage of disease content scores in videos.

With the exception of symptoms, videos received a majority score of one point for definitions, risk factors, assessment, management, and outcomes. In regard to symptoms, 78 (50%) of the video content received a score of two points, indicating that the majority of the videos fully refer to the clinical manifestations of the disease. Notably, a higher percentage of videos received a score of zero points for outcomes, management, and assessment, with 69 (45%), 57 (37%), and 41 (26%), respectively (Figure 1).

### Video Quality Assessments

The inter-observer reliabilities for the DISCERN, JAMA and GQS demonstrated excellent reliability, with ICC values of 0.973, 0.919, and 0.898 respectively. In this study, the median Total DISCERN scores, JAMA, and GQS of the 156 dengue-related videos under consideration were 26, 2, and 3, respectively. A comparative analysis was conducted on the scores of videos from various sources employing different rating scales. In DISCERN section 1, physicians and news agencies demonstrated significantly higher scores compared to individuals (*P*<.0001 and *P*<.01, respectively). Similarly, in DISCERN section 2, physicians exhibited significantly higher scores compared to individuals (*P*<.01). In DISCERN section 3, physicians’ scores were found to be significantly higher than both individuals’ and news agencies’ scores (*P*<.05). Additionally, the organization’s score was found to be significantly higher than individuals’ scores (*P*<.05). Furthermore, a statistically significant discrepancy was observed in the physician’s DISCERN total score when compared to that of the individuals (*P*<.001). Likewise, a significant disparity was noted in the DISCERN total score of the new agency when contrasted with that of the individuals (*P*<.05). A general trend indicates that the quality of dengue video information from professional sources is higher than that obtained from non-professional sources, particularly sources from individuals.

**Figure 2.**
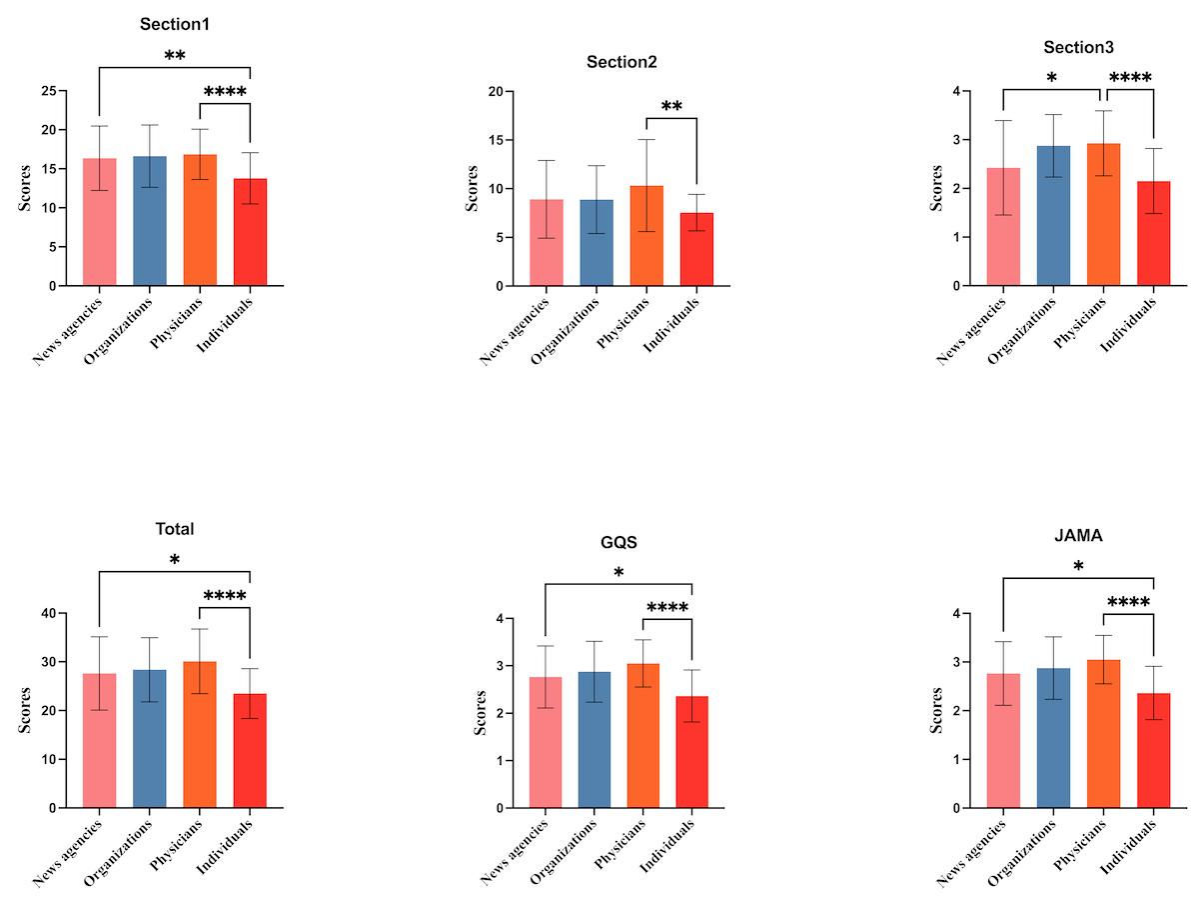
Quality score for Douyin videos of different sources. GQS: Global Quality Scale; JAMA: Journal of the American Medical Association. *P<.05, **P<.01, ***P<.001, ****P<.0001.

Moreover, the physician and news agency scores were found to be significantly higher than those of individuals with respect to GQS and JAMA scores (*P*<.05). In sum, the findings of the study demonstrated that physicians, organizations, and news agencies obtained consistently elevated scores when compared with individuals (see Figure 2).

### Correlation Analysis

A subsequent Spearman correlation analysis was conducted, revealing a positive correlation between video duration and DISCERN Section 1, DISCERN Section 3, DISCERN Total Score, and GQS (*r*=0.384, *P*<.001; *r*=0.279, *P*<.001; *r*=0.300, *P*<.001; and *r*=0.399, *P*<.001, respectively). In contrast, a negative correlation was observed between the following variables: video comments and DISCERN Section 1 (*r*=−0.163, *P*=.04), video comments and DISCERN Total Score (*r*=−0.222, *P*=.005), and video comments and JAMA (*r*=−0.229, *P*=.004). Furthermore, a negative correlation was identified between the number of days since posting and the following scores: DISCERN Section 1 (*r*=−0.192, *P*=.02), DISCERN Section 2 (*r*=−0.245, *P*<.002), DISCERN Total Score (*r*=−0.251, *P*=.002), and JAMA (*r*=−0.402, *P*<.001) (Table 3).

**Table 3.** Relationship between video basic characteristics and quality scores.

Basic characteristics	Section 1	Section 2	Section 3	Total	GQS^a^	JAMA^b^
Account fans						
*r* value	−0.071	−0.022	0.027	−0.046	0.041	0.110
*P* value	.38	.79	.74	.57	.61	.17
Account fans						
r value	−0.098	0.010	−0.006	−0.051	−0.001	0.094
*P* value	.22	.90	.94	.53	.99	.24
Video likes						
*r* value	−0.105	−0.067	−0.100	−0.130	−0.077	−0.147
*P* value	.19	.41	.21	.11	.34	.07
Video comment						
*r* value	−0.163	−0.139	−0.135	−0.222	−0.152	−0.229
*P* value	.04	.08	.09	.005	.06	.004
Video collections						
*r* value	−0.034	0.008	−0.065	−0.039	−0.005	−0.095
*P* value	.67	.92	.42	.63	.96	.24
Days since upload (days)						
*r* value	−0.192	−0.245	−0.028	−0.251	0.005	−0.402
*P* value	.02	.002	.73	.002	.95	.<001
Duration (seconds)						
*r* value	0.384	0.071	0.279	0.300	0.399	−0.001
*P* value	.<001	.38	.<001	.<001	.<001	.99

a GQS: Global Quality Scale.

b JAMA: Journal of the American Medical Association.

As demonstrated in Table S4 in Multimedia Appendix 1 clear linear relationship is evident between the video baseline features. The investigation revealed that the number of followers and the number of likes for the uploader were positively associated with the number of likes, comments, favorites, and posting time for the video. Furthermore, a positive correlation was observed between the number of likes, comments, and favorites for a video, while all three were positively correlated with posting time*;* all *Ps* <.01 (Table S4 in Multimedia Appendix 1).

## Discussion

### Principal Findings

TikTok, a popular short-form video platform, has amassed a substantial user base by offering innovative content, accurate recommendations, and social interaction features. Recent research has highlighted the potential of TikTok as a significant source of health information for consumers [25]. Dengue fever, an arboviral infection transmitted by mosquitoes, is endemic in tropical and subtropical regions. The dengue virus is classified into four distinct serotypes, and reinfection with different serotypes can lead to severe manifestations, including death [26]. This study was conducted for the purpose of analyzing videos pertaining to dengue fever that have been uploaded to the Chinese version of TikTok, which is known as Douyin. To assess the quality of the videos, the study utilized a variety of evaluation metrics, including DISCERN, GQS, and JAMA scores. In addition, the content of the videos was evaluated by focusing on six aspects of the disease. The videos related to dengue fever that were included in the study received a total of 718,228 likes and 126,400 comments, suggesting that Douyin has the potential to serve as a platform for disseminating health-related information and educating the public.

In this study, physicians were responsible for 81 (51%) of the dengue videos identified on Douyin, while individuals were responsible for 33 (21.2%) of the videos. However, it was observed that videos shared by individuals received more likes, comments, and favorites, which is consistent with previous research [27]. The following reasons are postulated as possible explanations for the observed phenomenon. First, videos from individuals may be more emotionally resonant or entertaining, and therefore more engaging. Second, individuals’ videos tend to use more colloquial language and vivid expressions that are easier to understand. Third, viewers are more likely to watch lighthearted and entertaining content than serious expertise, and videos from individuals are more in line with this preference. A salient feature pertains to the content scores, which reveal that the majority of the videos addressed the disease’s definition, symptoms, and risk factors; however, it is noteworthy that approximately one-quarter to one-half of the videos conspicuously omitted information concerning assessment, management, and outcomes. This phenomenon aligns with prior research, which revealed that between 12.2% and 44.4% of heatstroke videos on TikTok omitted crucial information regarding assessment, management, or outcomes [20]. Two potential explanations emerge. First, the brevity of Douyin videos, with an average duration of 86 seconds, poses challenges in addressing complex subjects such as disease assessment, management, and outcomes. However, the concise nature of the platform is conducive to the effective exposition of symptoms, definitions, and risk factors. Secondly, a considerable proportion of Douyin content creators are not healthcare professionals (with only 81 (51%) of content creators being physicians) and may not possess the expertise to thoroughly assess, manage, or report on disease-related outcomes. Moreover, the communication of this expertise in a comprehensible manner is also challenging.

This analysis reveals that the overall quality of videos on the Douyin platform is substandard, a finding that aligns with the conclusions of earlier research on gallstone disease videos on TikTok and orthodontic retention videos on the platform [28,29]. A significant discrepancy was identified between the highest and lowest scores obtained by physicians and individuals, respectively, in both the DISCERN section and the total scores when comparing the quality of videos from different sources. This observation was consistent across various evaluation metrics, including the GQS and JAMA scores. Fei Sun’s article revealed that individuals scored significantly lower than physicians in both the DISCERN section and the total scores [28]. Additionally, Nannan Cui et al article found that videos from physicians received higher GQS scores compared to videos from individuals [15]. A similar conclusion was reached in a study by Riccardo D’Ambrosi et al, who noted that the majority of videos exhibited low educational value, while videos posted by medical physicians exhibited higher educational value across all scores (JAMA, GQS, and DISCERN) [30]. Despite the fact that medical physicians achieved optimal results in comparison with other sources, it is important to note that the overall quality of the videos produced by medical physicians is not of a high standard. A preliminary analysis of the fundamental characteristics of the videos indicates that their brevity may be a contributing factor.

The findings of the study indicated a negative correlation between the temporal distance between video publication and analysis and quality, with the duration of the video exhibiting a positive correlation with quality. It is evident that the content of videos pertaining to medical issues may become outdated over time, particularly as medical knowledge undergoes rapid advancement. The information contained within older videos may become obsolete or no longer applicable, resulting in a deterioration in quality. Furthermore, shorter videos frequently exhibit deficiencies in both the quantity of information presented and the depth of their narrative, resulting in substandard production quality. These observations are consistent with the findings reported in previous studies [31]. A negative correlation has been demonstrated between the popularity of a video, especially its comments, and the video’s quality. Previous studies have indicated that unreliable videos exhibit higher user engagement metrics [32,33], findings that are consistent with the conclusions of this study. This phenomenon can be attributed to several factors. First, the presence of exaggerated or emotional content in low-quality videos is more likely to elicit an emotional response from the user, thereby increasing the number of comments. Second, the complexity of higher quality videos may require more time for users to process, thus leading to less frequent commenting. In contrast, lower quality videos are perceived as simple, prompting immediate and frequent reactions.

A positive correlation was identified among video likes, comments, and favorites, which are indicators of video popularity. In addition, the number of followers and likes of the uploader was found to be positively correlated with the other three variables. It is evident that content creators with a considerable number of followers typically possess a greater degree of influence, achieving a higher degree of content dissemination and a broader audience reach. Moreover, content creators who have amassed a significant following demonstrate a higher propensity to engage with their audience. Consequently, these creators are more likely to garner a greater number of likes, comments, and favorites on their videos. On the other hand, users tend to interact with videos that have already received a substantial number of likes, comments, and favorites. This phenomenon, known as the social proof effect, serves to further reinforce the positive correlation between these metrics.

The application of the DISCERN instrument revealed notably low scores in the two initial sections (reliability and treatment information), a phenomenon that is likely attributable to a fundamental misalignment between the tool’s design and the inherent characteristics of short-form visual media. The original development of DISCERN was for the purpose of evaluating comprehensive health information resources, and it emphasizes detailed assessments of evidence quality, alternative treatment options, and risk-benefit analyses. These elements are inherently constrained by the brevity and format limitations of short videos. While DISCERN remains a validated measure for assessing information quality in traditional formats, the findings of this study suggest that its conventional criteria may require modification to account for the unique communicative priorities of short-form platforms, where engagement and conciseness often supersede exhaustive detail. It is recommended that future adaptations of the scale consider incorporating metrics that are better suited to evaluating the accuracy and effectiveness of condensed health messaging.

The creation of a more extensive, widely viewed and high-quality corpus of disease knowledge videos will require the involvement of a greater number of healthcare professionals and specialized content creators. In addition, medical professionals and professional organizations should be incentivized to amass supporters through operating accounts and leverage the fan base to augment the dissemination of high-quality content. Concurrently, platforms should strengthen content review and quality control measures and recommend reliable, high-quality disease knowledge through optimized algorithms. In order to create high-quality videos, platforms need to support the development of videos with comprehensive content, extend the length of videos, and ensure timely content updates. Additionally, platforms should prioritize user education to promote scientific communication and disseminate health knowledge.

This study is subject to several limitations. Primarily, the content on Douyin is subject to rapid and continual updates, which may preclude the identification of long-term trends. Secondly, the evaluation of quality is inherently subjective. Thirdly, it is important to note that this study exclusively focused on the quality of content on the social media platform Douyin and did not consider other social media platforms or artificial intelligence.

### Conclusion

A cross-sectional study of dengue videos on Douyin revealed that medical professionals uploaded the highest number of videos; however, the overall quality was unsatisfactory. The number of followers of the creator is associated with the popularity of short videos, while the quality of video content is relatively weakly associated with dissemination. The health education information on Douyin is not rigorous enough to guide the public to make accurate judgments, as there are no experts to guide and supervise the accuracy of the content. In the current landscape, with the proliferation of self-media platforms and the advancements in artificial intelligence technology, there has been a significant surge in public access to health information. However, the quality of this information is highly variable, prompting the government to consider the implementation of a regulatory system, an audit mechanism, a professional certification system, and network communication standards. The implementation of these measures is intended to ensure the scientific integrity and accuracy of health information, to safeguard the public’s health rights and interests, and to establish a scientific and authoritative health knowledge dissemination system.

## Supplementary material

10.2196/76474 Multimedia Appendix 1 DISCERN scores, JAMA benchmark criteria, Global quality score, Pearson correlation analysis between basic characteristics of videos.

## Abbreviations

GQS: Global Quality Scale
ICC: intraclass correlation coefficients
JAMA: Journal of the American Medical Association

## References

[1] Wu T, Wu Z, Li YP (2022). Dengue fever and dengue virus in the People’s Republic of China. Rev Med Virol.

[2] Ly H (2024). Dengue fever in the Americas. Virulence.

[3] Tsheten T, Clements ACA, Gray DJ, Adhikary RK, Furuya-Kanamori L, Wangdi K (2021). Clinical predictors of severe dengue: a systematic review and meta-analysis. Infect Dis Poverty.

[4] Harapan H, Michie A, Sasmono RT, Imrie A (2020). Dengue: a minireview. Viruses.

[5] Yuya W, Yuansong Y, Susu L (2024). Progress and challenges in development of animal models for dengue virus infection. Emerg Microbes Infect.

[6] Velayudhan R, Control of Neglected Tropical Diseases (NTD), Viet Nam (2012). Global Strategy for Dengue Prevention and Control, 2012–2020 WHO.

[7] Wu JY, Lun ZR, James AA, Chen XG (2010). Dengue fever in mainland China. Am J Trop Med Hyg.

[8] Ng WL, Toh JY, Ng CJ (2023). Self-care practices and health-seeking behaviours in patients with dengue fever: a qualitative study from patients’ and physicians’ perspectives. PLoS Negl Trop Dis.

[9] Alyousefi TAA, Abdul-Ghani R, Mahdy MAK (2016). A household-based survey of knowledge, attitudes and practices towards dengue fever among local urban communities in Taiz Governorate, Yemen. BMC Infect Dis.

[10] Liu H, Fang CJ, Xu JW (2021). The health perceptions, dengue knowledge and control willingness among Dai ethnic minority in Yunnan Province, China. BMC Public Health.

[11] Lun X, Yang R, Lin L (2023). Effects of the source of information and knowledge of dengue fever on the mosquito control behavior of residents of border areas of Yunnan, China. Parasit Vectors.

[12] (2023). The 51st statistical report on the development status of the internet in China. China Internet Network Information Center (CNNIC).

[13] Moumane K, Idri AA World Conference on Information Systems and Technologies.

[14] Cui N, Lu Y, Cao Y, Chen X, Fu S, Su Q (2024). Quality assessment of TikTok as a source of information about mitral valve regurgitation in China: cross-sectional study. J Med Internet Res.

[15] Ostrovsky AM, Chen JR (2020). TikTok and its role in COVID-19 information propagation. J Adolesc Health.

[16] Yeung A, Ng E, Abi-Jaoude E (2022). TikTok and attention-deficit/hyperactivity disorder: a cross-sectional study of social media content quality. Can J Psychiatry.

[17] Baumel NM, Spatharakis JK, Karitsiotis ST, Sellas EI (2021). Dissemination of mask effectiveness misinformation using TikTok as a medium. J Adolesc Health.

[18] Zhang Y, Sun Y, Xie B (2015). Quality of health information for consumers on the web: a systematic review of indicators, criteria, tools, and evaluation results. Asso for Info Science & Tech.

[19] Śledzińska P, Bebyn MG, Furtak J (2021). Quality of YouTube videos on meningioma treatment using the DISCERN instrument. World Neurosurg.

[20] Qiu J, Zhou YL (2024). Quality assessment of heatstroke videos on TikTok. Front Public Health.

[21] Jiang SS, Zhou YL, Qiu J, Gou XH (2025). Search engines and short video apps as sources of information on acute pancreatitis in China: quality assessment and content assessment. Front Public Health.

[22] Yacob M, Lotfi S, Tang S, Jetty P (2020). Wikipedia in vascular surgery medical education: comparative study. JMIR Med Educ.

[23] Du RC, Zhang Y, Wang MH, Lu NH, Hu Y (2023). TikTok and Bilibili as sources of information on Helicobacter pylori in China: A content and quality analysis. Helicobacter.

[24] Goobie GC, Guler SA, Johannson KA, Fisher JH, Ryerson CJ (2019). YouTube videos as a source of misinformation on idiopathic pulmonary fibrosis. Ann Am Thorac Soc.

[25] Song S, Zhao YC, Yao X, Ba Z, Zhu Q (2022). Serious information in hedonic social applications: affordances, self-determination and health information adoption in TikTok. Journal of Documentation.

[26] Kularatne SA, Dalugama C (2022). Dengue infection: global importance, immunopathology and management. Clin Med (Lond).

[27] Zheng S, Tong X, Wan D, Hu C, Hu Q, Ke Q (2023). Quality and reliability of liver cancer-related short Chinese videos on TikTok and Bilibili: cross-sectional content analysis study. J Med Internet Res.

[28] Sun F, Zheng S, Wu J (2023). Quality of information in gallstone disease videos on TikTok: cross-sectional study. J Med Internet Res.

[29] Meade MJ, Dreyer CW (2022). Analysis of the information contained within TikTok videos regarding orthodontic retention. J World Fed Orthod.

[30] D’Ambrosi R, Bellato E, Bullitta G (2024). TikTok and frozen shoulder: a cross-sectional study of social media content quality. J Orthop Traumatol.

[31] Gong X, Dong B, Li L, Shen D, Rong Z (2023). TikTok video as a health education source of information on heart failure in China: a content analysis. Front Public Health.

[32] Warren CJ, Sawhney R, Shah T, Behbahani S, Sadeghi-Nejad H (2021). YouTube and men’s health: a review of the current literature. Sex Med Rev.

[33] Mueller SM, Hongler VNS, Jungo P (2020). Fiction, falsehoods, and few facts: cross-sectional study on the content-related quality of atopic eczema-related videos on YouTube. J Med Internet Res.

