# Correction: Quality Assessment of Videos About Dengue Fever on Douyin: Cross-Sectional Study

**DOI:** 10.2196/85305

**Published:** 2025-10-20

**Authors:** Youlian Zhou, Liang Yang, Li Luo, Lianghai Cao, Jun Qiu

**Affiliations:** 1 Intensive Care Unit, Chengdu Integrated TCM & Western Medicine Hospital, 18 North Vientiane Road, Chengdu, 610016, China, 86 18380215259

In “Quality Assessment of Videos About Dengue Fever on Douyin: Cross-Sectional Study” [1], the authors noted two errors.

In the reference list, two references were incorrect and two references were missing. The in-text numeration is correct and remains unchanged, but the numeration within the reference list has been adjusted accordingly.

The following references, previously listed as references 13 and 25, were removed:

*Al-Badareen AB, Selamat MH, Jabar MA. Software Quality Models: A Comparative Study. Springer; 2011. [doi: 10. 1007/978-3-642-22170-5_4]*

*Bernard A, Langille M, Hughes S, Rose C, Leddin D, Veldhuyzen van Zanten S. A systematic review of patient inflammatory bowel disease information resources on the World Wide Web. Am J Gastroenterol. Sep 2007;102(9):2070-2077. [doi: 10.1111/j.1572-0241.2007.01325.x] [Medline: 17511753]*

In addition, the following references have been added as references 30 and 31:

*D'Ambrosi R, Bellato E, Bullitta G, et al. TikTok and frozen shoulder: a cross-sectional study of social media content quality. J Orthop Traumatol. Nov 24, 2024;25(1):57. [doi: 10.1186/s10195-024-00805-y] [Medline: 39581922]*

*Gong X, Dong B, Li L, Shen D, Rong Z. TikTok video as a health education source of information on heart failure in China: a content analysis. Front Public Health. Dec 2023;11(11):1315393. [doi: 10.3389/fpubh.2023.1315393] [Medline: 38146471]*

The reference list numeration has been adjusted accordingly, and in-text citation numeration remains unaffected.

The correction will appear in the online version of the paper on the JMIR Publications website, together with the publication of this correction notice. Because this was made after submission to PubMed, PubMed Central, and other full-text repositories, the corrected article has also been resubmitted to those repositories.
