# Supplementary material for: Quality Assessment of Videos About Dengue Fever on Douyin: Cross-Sectional Study
Source: JMIR Infodemiology. 2025 Sep 26;5:e76474. doi: 10.2196/76474 (PMC12466789; doi:10.2196/76474)
Supplement: Multimedia Appendix 1 [file infodemiology-v5-e76474-s001.docx]

(Supplementary table1): The JAMA Benchmark Criteria

| Authorship | Authors and contributors, their affiliations, and relevant credentials should be provided. |
| --- | --- |
| Attribution | References and sources for all content should be listed clearly, and all relevant copyright information noted. |
| Disclosure | Web site "ownership" should be prominently and fully disclosed, as should any sponsorship, advertising, underwriting, commercial funding arrangements or support, or potential conflicts of interest. This includes arrangements in which links to other sites are posted as a result of financial considerations. Similar standards should hold in discussion forums. |
| Currency | Dates that content was posted and updated should be indicated. |

(Supplementary table2): GQS（Global quality Score）

| 1 | Poor quality,poor flow of the site,most information missing,not at all useful for patients |
| --- | --- |
| 2 | Generally poor quality and poor flow, some information listed but many important topics missing, of very limited use to patients |
| 3 | Moderate quality, suboptimal flow, some important information is adequately discussed but others poorly discussed, somewhat useful for patient |
| 4 | Good quality and generally good flow, most of the relevant information is listed, |
| 5 | Excellent quality and excellent flow, very useful for patients |

(Supplementary table3): The DISCERN scores

| Section1: DISCERN SCORE |
| --- |
| 1. Are the aims clear? |
| 1. Does it achieve its aims? |
| 1. Is it relevant? |
| 1. Is it clear what sources of information were used to compile the publication (other than the author or producer)? |
| 1. Is it clear when the information used or reported in the publication was produced? |
| 1. Is it balanced and unbiased? |
| 1. Does it provide details of additional sources of support and information? |
| 1. Does it refer to areas of uncertainty? |
| Section2: HOW GOOD IS THE QUALITY OF INFORMATION ON TREATMENT CHOICES? |
| 1. Does it describe how each treatment works? |
| 1. Does it describe the benefits of each treatment? |
| 1. Does it describe the risks of each treatment? |
| 1. Does it describe what would happen if no treatment were used? |
| 1. Does it describe how the treatment choices affect overall quality of life? |
| 1. Is it clear that there may be more than one possible treatment choice? |
| 1. does it provide support for shared decision-making? |
| Section 3: OVERALL RATING OF THE PUBLICATION |
| 1. Based on the answers to all the above questions, rate the overall quality of the publication as a source of information about treatment choices: |

**Supplementary table4: Pearson correlation analysis between basic characteristics of Videos**

|  | Account fans | Account  likes | Video  likes | Video comment | Video collections | Days since upload (days) | Duration (seconds) |
| --- | --- | --- | --- | --- | --- | --- | --- |
| Account fans |  |  |  |  |  |  |  |
| r value | 1.000 | .967^**^ | .599^**^ | .398^**^ | .561^**^ | .215^**^ | -0.011 |
| P value | - | 0.000 | 0.000 | 0.000 | 0.000 | 0.007 | 0.895 |
| Account likes |  |  |  |  |  |  |  |
| r value | .967^**^ | 1.000 | .608^**^ | .405^**^ | .559^**^ | .209^**^ | -0.088 |
| P value | 0.000 | - | 0.000 | 0.000 | 0.000 | 0.009 | 0.275 |
| Video likes |  |  |  |  |  |  |  |
| r value | .599^**^ | .608^**^ | 1.000 | .893^**^ | .963^**^ | .402^**^ | 0.019 |
| P value | 0.000 | 0.000 | - | 0.000 | 0.000 | 0.000 | 0.818 |
| Video comment |  |  |  |  |  |  |  |
| r value | .398^**^ | .405^**^ | .893^**^ | 1.000 | .862^**^ | .434^**^ | -0.028 |
| P value | 0.000 | 0.000 | 0.000 | - | 0.000 | 0.000 | 0.731 |
| Video collections |  |  |  |  |  |  |  |
| r value | .561^**^ | .559^**^ | .963^**^ | .862^**^ | 1.000 | .370^**^ | 0.022 |
| P value | 0.000 | 0.000 | 0.000 | 0.000 | - | 0.000 | 0.784 |
| Days since upload (days) |  |  |  |  |  |  |  |
| r value | .215^**^ | .209^**^ | .402^**^ | .434^**^ | .370^**^ | 1.000 | 0.025 |
| P value | 0.007 | 0.009 | 0.000 | 0.000 | 0.000 | - | 0.758 |
| Duration (seconds) |  |  |  |  |  |  |  |
| r value | -0.011 | -0.088 | 0.019 | -0.028 | 0.022 | 0.025 | 1.000 |
| P value | 0.895 | 0.275 | 0.818 | 0.731 | 0.784 | 0.758 | - |

| -not applicable.  *P<.05  **P<.01 |
| --- |
